# A Case of Pleomorphic-Type Anaplastic Carcinoma of the Pancreas with Rapidly Progressive and Fatal Cardiac Metastasis

**DOI:** 10.70352/scrj.cr.25-0567

**Published:** 2026-01-07

**Authors:** Keiichi Shigematsu, Shingo Kozono, Atsushi Fujii, Norimasa Abe, Hirotaka Kuga, Sadafumi Tamiya, Toru Nakano

**Affiliations:** 1 Department of Surgery, Kitakyushu Municipal Medical Center, Kitakyushu, Fukuoka, Japan; 2 Department of Pathology, Kitakyushu Municipal Medical Center, Kitakyushu, Fukuoka, Japan

**Keywords:** pancreatic ductal adenocarcinoma, cardiac metastasis, pleomorphic-type anaplastic carcinoma of the pancreas, undifferentiated carcinoma of the pancreas

## Abstract

**INTRODUCTION:**

Cardiac metastasis from pancreatic ductal adenocarcinoma (PDAC) is extremely rare. Pleomorphic-type anaplastic carcinoma of the pancreas (PACP) is an uncommon, highly aggressive PDAC variant with lymphatic and hematogenous spread and poor prognosis. We report, to our knowledge, the first PACP case with antemortem diagnosis of cardiac metastasis and a rapidly fatal course.

**CASE PRESENTATION:**

A 75-year-old man presented with left hypochondrial pain. CT showed a 4-cm, gradually and heterogeneously enhancing pancreatic tail mass without distant metastasis. Gadolinium-enhanced MRI demonstrated peripheral progressive enhancement with internal non-enhancing areas, suggesting a non-conventional PDAC. Ultrasound-guided fine needle aspiration (EUS-FNA), nevertheless, yielded mainly scattered atypical epithelial cells with a small cohesive columnar component, and adenocarcinoma was diagnosed; further subclassification was not feasible on the limited cytologic material. Because the tumor was radiologically resectable, neoadjuvant gemcitabine plus S-1 was administered. Follow-up CT showed enlargement to 5.5 cm, new invasion of the spleen and gastric wall, and peripheral progressive enhancement—previously demonstrated on MRI—now appreciable on CT, while distant disease remained absent. Distal pancreatectomy with splenectomy, regional lymphadenectomy, and partial gastrectomy was performed. The resected specimen revealed a whitish solid tail tumor with focal hemorrhage/necrosis; histology confirmed PACP. Adjuvant S-1 was initiated 1 month postoperatively. Three months after surgery, CA19-9 increased despite no recurrence on CT. One month later he presented with exertional dyspnea and complete atrioventricular block. Echocardiography and CT showed an approximately 8-cm interatrial mass and a left-ventricular wall lesion. Retrospective review of CT performed 1 month earlier identified a 2-cm atrial nodule at the same site, indicating very rapid intracardiac growth. He was transferred to a cardiovascular center for planned pacemaker implantation, and heart-failure therapy was initiated first. During preparation he developed sudden cardiac arrest and died. Postmortem myocardial biopsy confirmed PACP metastasis.

**CONCLUSIONS:**

Clinicians need to recognize that PACP can metastasize to the heart and incorporate this risk into routine follow-up, maintaining vigilance for imaging features suggestive of cardiac involvement and for new-onset cardiac symptoms or conduction disturbances; when cardiac metastasis is suspected, prompt evaluation and intervention are warranted.

## Abbreviations

CA19-9
: carbohydrate antigen 19-9
CEA
: carcinoembryonic antigen
CRP
: C-reacitive protein
EUS-FNA
: ultrasound-guided fine needle aspiration
NAC
: neoadjuvant chemotherapy
PACP
: pleomorphic-type anaplastic carcinoma of the pancreas
PDAC
: pancreatic ductal adenocarcinoma
WBC
: white blood cell

## INTRODUCTION

Cardiac metastasis from malignant tumors has been reported in 2.3%–18.3% of autopsy cases.^1,2)^ However, cardiac involvement from gastrointestinal cancers is relatively uncommon, and in particular, cardiac metastasis from pancreatic ductal adenocarcinoma (PDAC) is extremely rare.^1–3)^ Pleomorphic anaplastic carcinoma of the pancreas (PACP) is a rare histologic subtype of pancreatic cancer, classified as “undifferentiated carcinoma” in the 5th edition of the WHO classification.^4)^ It is characterized by expansile growth and a strong tendency to metastasize via hematogenous and lymphatic routes.^5,6)^ This subtype is known to have a poorer prognosis compared with conventional PDAC.^6,7)^ Tschang et al. conducted an autopsy study of 15 cases of pleomorphic carcinoma of the pancreas and reported cardiac metastasis in 5 of 15 cases.^8)^ Previous reports have suggested that cardiac metastasis is relatively common in autopsy series, yet to our knowledge, no cases of PACP with cardiac involvement diagnosed antemortem have been reported. In the present report, we describe, for the first time, a case of PACP in which cardiac metastasis was detected 4 months after curative resection and rapidly progressed to a fatal outcome.

## CASE PRESENTATION

**Fig. 1:**
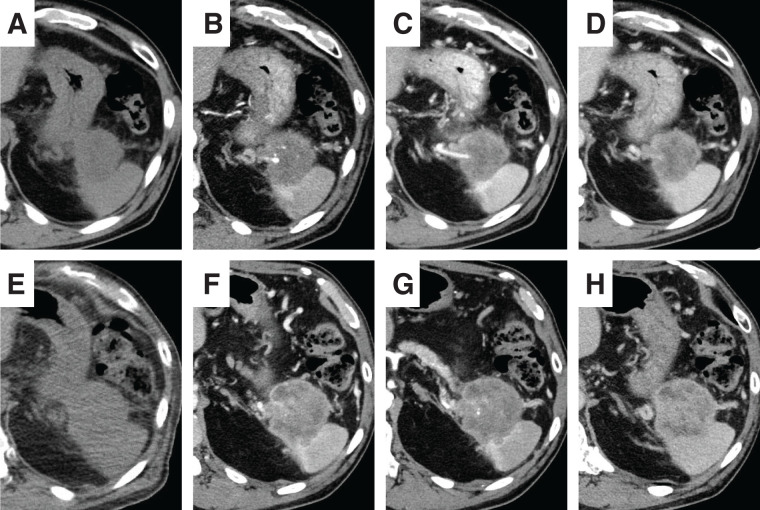
Contrast-enhanced CT before and after neoadjuvant chemotherapy. (**A**–**D**) Initial CT. A 4-cm mass is seen in the pancreatic tail, showing gradual and heterogeneous enhancement. (**E**–**H**) CT after completion of NAC-GS. The mass has enlarged to 5.5 cm and shows invasion of the adjacent spleen and gastric wall. The tumor now exhibits more conspicuous peripheral enhancement with scattered intratumoral low attenuation areas, findings that may reflect tumor degeneration, including focal necrosis, secondary to rapid enlargement. (**A**, **E**) Unenhanced phase; (**B**, **F**) arterial phase; (**C**, **G**) portal venous phase; (**D**, **H**) equilibrium phase. NAC-GS, neoadjuvant gemcitabine plus S-1

**Fig. 2:**
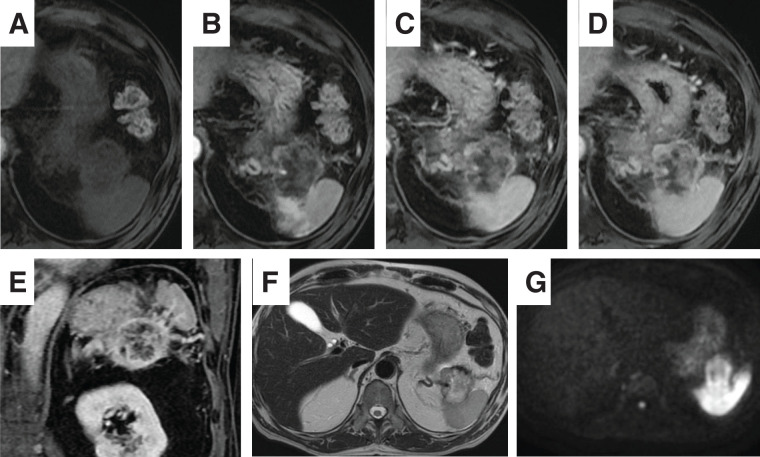
MRI of the pancreatic tail mass before NAC-GS. (**A**) Precontrast fat-suppressed T1-weighted image shows a low-signal mass in the pancreatic tail. (**B**–**D**) Dynamic gadolinium-enhanced fat-suppressed T1-weighted images demonstrate progressive peripheral enhancement from the early arterial (**B**) through the portal venous (**C**) to the delayed phase (**D**). Within the tumor, scattered non-enhancing low-signal areas persist throughout these phases, corresponding to intratumoral degeneration, including focal necrosis, associated with rapid tumor enlargement, as suggested on CT. (**E**) Coronal gadolinium-enhanced T1-weighted image again shows peripheral enhancement with internal low-signal areas. (**F**) T2-weighted image shows a mildly hyperintense, mosaic-like pattern. (**G**) Diffusion-weighted image shows high signal within the lesion. NAC-GS, neoadjuvant gemcitabine plus S-1

**Fig. 3:**
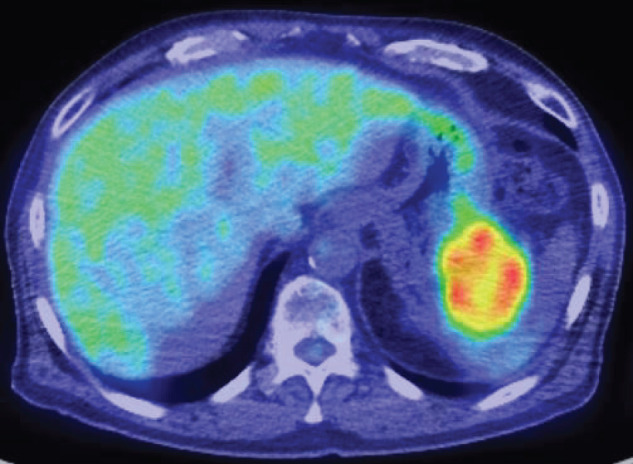
PET-CT of the pancreatic tail tumor before NAC-GS. Fused PET-CT demonstrates a 5.5-cm heterogeneously FDG-avid mass in the pancreatic tail (SUVmax = 6.46). The central portion of the lesion exhibits reduced FDG uptake, suggesting intratumoral necrosis. No abnormal FDG accumulation indicating lymph node, peritoneal, or distant metastasis is observed. NAC-GS, neoadjuvant gemcitabine plus S-1; SUVmax, maximum standardized uptake value

**Fig. 4:**
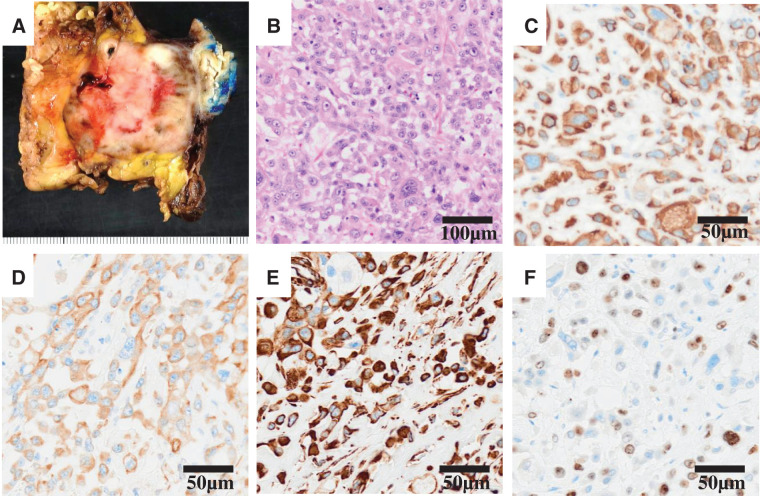
Histopathological and immunohistochemical findings of the resected specimen. (**A**) Macroscopically, the tumor is a whitish solid mass with areas of hemorrhage and yellowish degenerative change consistent with tumor necrosis. (**B**) Hematoxylin and eosin staining shows a poorly differentiated carcinoma composed of pleomorphic mononuclear cells admixed with bizarre multinucleated giant cells without glandular formation. (**C**) Immunohistochemistry for pan-cytokeratin (AE1/AE3) shows diffuse cytoplasmic positivity in the atypical tumor cells, confirming their epithelial nature. (**D**) Cytokeratin 19 is diffusely positive, supporting pancreatic ductal epithelial differentiation. (**E**) Tumor cells coexpress vimentin, indicating sarcomatoid or epithelial–mesenchymal transition–like features characteristic of pleomorphic/anaplastic pancreatic ductal carcinoma. (**F**) MIB-1 (Ki-67) labeling index is approximately 40%, reflecting high proliferative activity.

A 75-year-old man was referred to our hospital due to a pancreatic tail mass detected during evaluation for left hypochondrial pain. He had no family history of malignancy. Initial laboratory tests revealed no inflammatory signs (WBC count: 6130/μL, CRP: 0.063 mg/dL), but elevated CA19-9 levels at 1949.6 U/mL, while CEA remained normal at 2.7 ng/mL. Initial contrast enhanced abdominal CT demonstrated a 4-cm, irregular, gradually and heterogeneously enhancing mass in the pancreatic tail, without regional lymphadenopathy or distant metastasis (**Fig. 1A**–**1D**). Based on CT alone, a conventional PDAC was primarily suspected. However, contrast-enhanced MRI demonstrated more clearly than CT a pattern of peripheral progressive enhancement with persistent internal non-enhancing areas, a finding that was less conspicuous on CT and somewhat atypical for conventional PDAC; therefore, other pancreatic tumors such as PACP, adenosquamous carcinoma, pancreatic neuroendocrine tumor with degenerative change, or solid pseudopapillary neoplasm were also taken into account (**Fig. 2**). PET-CT showed FDG uptake confined to the pancreatic tail lesion and no extra-pancreatic disease (**Fig. 3**). Ultrasound-guided fine needle aspiration (EUS-FNA) yielded predominantly singly scattered atypical cells with a focal cohesive, columnar epithelial component, so pancreatic adenocarcinoma was presumed. Nevertheless, because the aspirated material was limited, the proportion of poorly or undifferentiated components could not be evaluated, and definitive histologic subtyping was not possible at this stage. On the basis of the cytologic diagnosis of adenocarcinoma and the resectable imaging status, neoadjuvant chemotherapy with gemcitabine plus S-1 (NAC-GS) was initiated. After NAC-GS, CA19-9 levels increased to 2294.7 U/mL, and follow-up CT showed tumor enlargement to 5.5 cm, new invasion of the adjacent spleen and gastric wall, and, notably, the peripheral enhancement with scattered intratumoral low-attenuation areas that had been more apparent on MRI before NAC now became evident also on CT (**Fig. 1E**–**1H**). This temporal change further suggested that the lesion might not represent a conventional PDAC. Even so, there were still no findings of unresectability such as liver or lung metastasis, peritoneal dissemination, or major arterial encasement. Because disease progression appeared to be confined to the primary site, and the patient wished to proceed, surgical resection was planned. Surgical exploration revealed no liver metastasis or peritoneal dissemination, and peritoneal lavage cytology was negative. Therefore, standard distal pancreatectomy with regional lymph node dissection was performed. The tumor infiltrated the posterior wall of the upper gastric body, necessitating partial gastrectomy. On gross examination, the resected specimen showed a whitish, solid mass in the pancreatic tail with areas of hemorrhage and yellowish degenerative change consistent with tumor necrosis (**Fig. 4A**). Histologically, the tumor was a poorly differentiated carcinoma composed of pleomorphic mononuclear atypical cells admixed with bizarre multinucleated giant cells, with virtually no glandular formation, leading to a diagnosis of pleomorphic-type anaplastic carcinoma of the pancreas (**Fig. 4B**). Immunohistochemically, the tumor cells were diffusely positive for pan-cytokeratin (AE1/AE3) and cytokeratin 19, confirming pancreatic ductal epithelial differentiation, and they coexpressed vimentin, indicating sarcomatoid/epithelial–mesenchymal transition-like features characteristic of this variant. The MIB-1 (Ki-67) labeling index was approximately 40%, indicating high proliferative activity (**Fig. 4C**–**4F**). In all, 2 out of 39 dissected lymph nodes were positive for metastasis. Final pathological stage was T3N2M0, stage III. Postoperative CA19-9 decreased to 53.5 U/mL, and adjuvant chemotherapy with S-1 was initiated 1 month postoperatively.

**Fig. 5:**
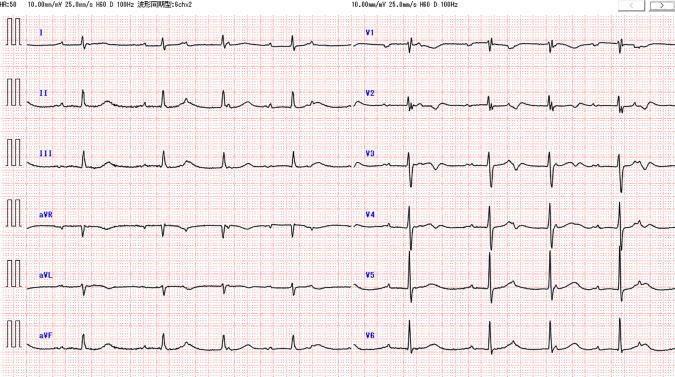
Electrocardiogram findings. The electrocardiogram reveals a heart rate of 50 beats per minute and 3rd-degree atrioventricular block.

**Fig. 6:**
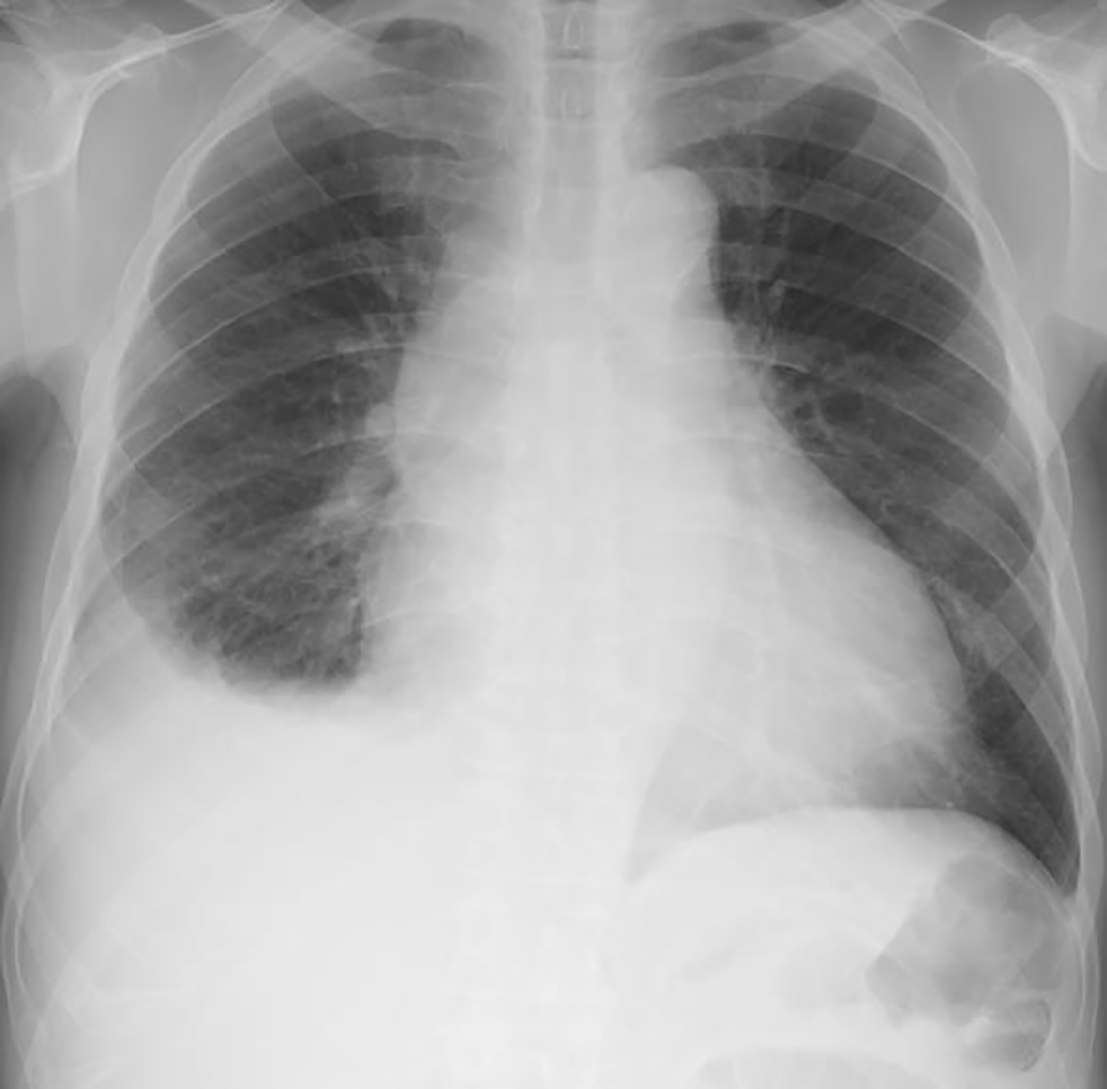
Chest X-ray imaging. Cardiomegaly, enhanced pulmonary hilar shadows, and right-sided pleural effusion are observed.

**Fig. 7:**
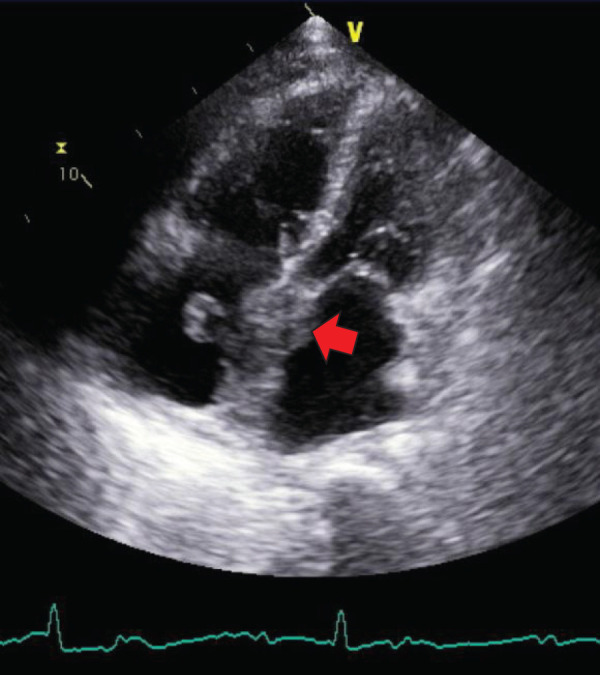
Echocardiogram imaging. Wall thickening with irregularities in the atrial septum (arrow) suggests a tumor-like lesion.

**Fig. 8:**
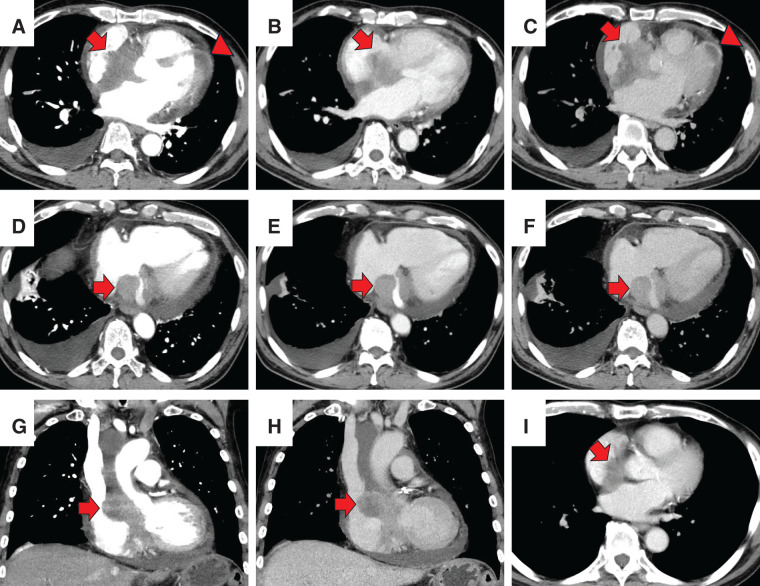
Contrast-enhanced chest CT demonstrating cardiac metastases. (**A**–**C**) Axial images show an 8-cm heterogeneously enhancing mass centered on the interatrial septum (arrow) and a 2-cm enhancing nodule on the free wall of the left ventricle (arrowhead). (**D**–**F**) At a slightly caudal level, tumoral proliferation is seen around the coronary sinus with resultant narrowing of the venous lumen (arrows). (**G**, **H**) Coronal reformatted images confirm a tumor along the interatrial septum (arrows). (**I**) On retrospective review of CT performed 1 month earlier, a 2-cm enhancing lesion on the right side of the interatrial septum can be identified (arrow). (**A**, **D**, **G**) arterial phase; (**B**, **E**, **I**) portal venous phase; (**C**, **F**, **H**) equilibrium phase.

Three months after surgery, CA19-9 levels increased to 86.3 U/mL. CT scans revealed no obvious recurrence, and the patient continued S-1 chemotherapy. However, 1 month later, the patient presented to our emergency department with symptoms of exertional dyspnea and irregular pulse. Upon admission, his body temperature was 36.9°C, blood pressure was 143/64 mmHg, pulse rate was 52 bpm, and SpO_2_ was 94% on room air. Laboratory findings indicated mild inflammation (WBC count: 9460/μL, CRP: 3.7 mg/dL), elevated BNP at 574.0 pg/mL, and markedly elevated CA19-9 at 400.8 U/mL. ECG revealed 3rd-degree atrioventricular block with a heart rate of 50 bpm (**Fig. 5**). Chest X-ray showed cardiomegaly (CTR 55%) and right pleural effusion (**Fig. 6**). Echocardiography demonstrated thickened atrial septum suspicious of a tumorous lesion and another lesion in the left ventricular apex (**Fig. 7**). Contrast-enhanced CT then detected an 8-cm heterogeneously enhancing mass centered on the interatrial septum, causing narrowing of the coronary sinus, together with a 2-cm enhancing lesion in the left ventricular wall (**Fig. 8A**–**8H**). Retrospective review of CT performed 1 month earlier revealed that a 2-cm enhancing lesion in the interatrial septum had already been present but was smaller, indicating rapid cardiac progression (**Fig. 8I**). Clinical findings and elevated CA19-9 strongly suggest metastatic cardiac tumor from PACP. The patient was referred to a tertiary cardiovascular center for evaluation and management of heart failure symptoms and complete AV block. Transvenous pacing was considered high-risk due to interference with the right atrial mass, so initial management included diuretics for heart failure started. Although plans had been made for intracardiac biopsy and pacing, or alternatively epicardial pacing via mini thoracotomy, the patient unfortunately experienced sudden cardiac arrest 3 days after transfer before these procedures could be performed. Resuscitation attempts were unsuccessful, and he died 4 months after the pancreatic surgery. ECG monitoring prior to cardiac arrest showed short runs of non-sustained ventricular tachycardia, complete AV block, and ST elevation. Postmortem myocardial biopsy from the left ventricular apex confirmed cardiac metastasis from PACP.

## DISCUSSION

We report the first known case of PACP with cardiac metastasis diagnosed antemortem. The disease followed an aggressive clinical course, culminating in sudden death. Metastatic cardiac tumors are approximately 40–100 times more common than primary cardiac tumors.^3,9)^ Metastatic cardiac tumors are identified in 2.3%–18.3% of autopsy cases involving malignancy.^1,2)^ The most frequent primary sources include lung cancer, hematologic malignancies, breast cancer, and esophageal cancer, with pancreatic cancer accounting for 3.6%–6.4%.^2,10)^ While cardiac metastases from pancreatic cancer are occasionally detected at autopsy, antemortem diagnosis is exceedingly rare. PDAC commonly metastasizes to the liver, peritoneum, lungs, bones, or distant lymph nodes.^11)^ Cardiac metastasis typically reflects advanced systemic disease and rarely presents with isolated cardiac symptoms.^3)^ In the present case, exertional dyspnea prompted further evaluation, leading to the detection of cardiac metastasis. Symptomatic cardiac metastasis from PDAC resulting in an antemortem diagnosis is exceptionally rare. This case therefore represents a highly unusual and clinically significant presentation.

The manifestations of cardiac metastases depend on the anatomical site involved.^1,2)^ Pericardial infiltration may lead to pericardial effusion and tamponade. Myocardial involvement can cause conduction abnormalities and arrhythmias, including atrial fibrillation, ventricular ectopy, complete atrioventricular (AV) block, or acute myocardial infarction due to coronary artery obstruction. This case developed exertional dyspnea and a pulse deficit, which were attributed to heart failure and complete AV block caused by myocardial invasion. Pacemaker implantation was planned; however, the patient experienced sudden cardiac death before the procedure could be performed. PACP is characterized by rapid and expansile tumor growth.^5,6,12)^ In this case, tumor infiltration into the interatrial septum and ventricular myocardium likely disrupted the cardiac conduction system and caused myocardial ischemia, ultimately leading to a fatal outcome.

Cardiac metastases may occur via four principal routes: (1) hematogenous dissemination, (2) lymphatic spread, (3) direct extension, and (4) transvenous extension via the vena cava.^2)^ In the present case, the metastatic lesion was primarily located in the interatrial septum, suggesting hematogenous spread. Four previously reported cases of pancreatic cancer with cardiac metastases similarly involved the right atrium or right ventricle and showed no evidence of mediastinal lymphadenopathy, further supporting hematogenous dissemination as the likely route.^13–16)^

Management of cardiac metastases consists of both tumor-directed and symptom-directed strategies.^1–3,9,17)^ Symptomatic treatment may include medical management of heart failure or pacemaker implantation. In selected cases, surgical resection can alleviate cardiac obstruction; however, this approach is generally limited to situations in which systemic disease is well controlled.^18,19)^ In PDAC, cardiac metastasis is typically observed as part of systemic disease progression and therefore is rarely considered a target for surgical resection. Only one case has been reported in which cardiac metastasis was surgically resected following surgery for PDAC.^20)^ The lesion was discovered 6 weeks postoperatively after the patient developed pulmonary artery thrombosis. A 8-cm tumor was identified in the right atrium, prolapsing into the right ventricle and obstructing the tricuspid valve. The patient underwent surgical resection of the cardiac lesion and recovered sufficiently to receive postoperative chemotherapy. The patient ultimately died 9 months after surgery. In two other reported cases, surgical resection was not performed; instead, systemic chemotherapy was administered, resulting in tumor shrinkage of cardiac metastases and temporary disease control.^14,16)^ Therefore, systemic chemotherapy is typically considered as the first-line treatment for cardiac metastases, particularly in patients with mild or manageable symptoms. Considering the findings of these case reports, surgical resection of cardiac metastasis may be beneficial in carefully selected patients, particularly when it provides symptom relief and enables the continuation of systemic therapy.

PACP is a rare subtype of pancreatic cancer.^7,21)^ Anaplastic pancreatic carcinoma was first reported by Sommers et al. in 1954 as pleomorphic carcinoma with sarcomatoid components.^22)^ It accounts for approximately 2%–7% of all pancreatic malignancies.^7,21)^ According to the 5th edition of the WHO classification, anaplastic carcinoma is categorized under undifferentiated carcinoma and is subdivided into undifferentiated carcinoma (UC)—comprising anaplastic UC, sarcomatoid UC, and carcinosarcoma—and undifferentiated carcinoma with osteoclast-like giant cells (UCOGC).^4)^ The present case falls into the category of anaplastic UC. PACP often grows expansively, forming large, hypervascular tumors.^5,6,8,12)^ On CT, PACP typically shows heterogeneous enhancement with central necrosis and/or cystic change and rapid interval growth, unlike the relatively homogeneous hypoenhancing appearance of conventional PDAC, yet CT is not discriminatory.^23–25)^ EUS-FNA confirms malignancy but often underperforms for undifferentiated variants because limited sampling lacks architecture and tends to capture only the ductal component, leading to preoperative misclassification as conventional PDAC. While EUS-FNA can diagnose UC in some reports,^26–28)^ other series show low preoperative cytologic accuracy underscoring the limitations of FNA alone for reliable subtype classification.^12,29)^ Adjunctive measures—including EUS-FNB,^30)^ contrast-enhanced EUS to target viable (non-necrotic) areas,^31)^ and rapid on-site evaluation (ROSE)^32)^—can improve diagnostic adequacy, although PACP-specific evidence remains limited. PACP tends to metastasize early via both hematogenous and lymphatic routes and demonstrates rapid progression, resulting in a poorer prognosis compared with conventional PDAC.^6,7,33)^ In this case as well, the cardiac metastasis enlarged rapidly and was associated with a fatal clinical course, reflecting the high proliferative potential of PACP. Tschang et al. analyzed 15 autopsy cases of pancreatic pleomorphic carcinoma and reported metastatic sites as the liver (87%), lung (73%), adrenal gland (60%), kidney (47%), bone (40%), heart (33%), and thyroid (20%).^8)^ These findings suggest that cardiac metastasis, as observed in our patient, is not exceedingly rare in PACP, and clinicians should remain vigilant for cardiac involvement in this disease. Although PACP is considered to have a poorer prognosis than PDAC, it has been reported that patients undergoing curative resection for PACP can achieve improved outcomes, with survival comparable to that of PDAC.^12,21)^ As for chemotherapy, no standard regimen has yet been established for PACP. However, regimens containing paclitaxel have recently shown promising tumor control, and the combination of nab-paclitaxel with gemcitabine has been reported to be effective.^34)^ In the present case, retrospective review of CT images obtained at the time when recurrence was first suspected revealed findings suggestive of cardiac metastasis. Had cardiac involvement been recognized at that point, earlier diagnosis and timely initiation of chemotherapy with a nab-paclitaxel plus gemcitabine regimen might have been possible—potentially prolonging the patient’s survival.

## CONCLUSIONS

We report a rare case of PACP with an antemortem diagnosis of cardiac metastasis, resulting in rapid and fatal progression. Clinicians should recognize the potential for cardiac metastasis and consider early intervention.
